# Exercise and Dietary-Protein as a Countermeasure to Skeletal Muscle Weakness: Liverpool Hope University – Sarcopenia Aging Trial (LHU-SAT)

**DOI:** 10.3389/fphys.2019.00445

**Published:** 2019-04-25

**Authors:** Ben Kirk, Kate Mooney, Farzad Amirabdollahian, Omid Khaiyat

**Affiliations:** School of Health Sciences, Liverpool Hope University, Liverpool, United Kingdom

**Keywords:** aging, muscle weakness, exercise, dietary-protein, leucine

## Abstract

**Objective:**

To investigate the effects of a 16-week concurrent exercise regimen [resistance exercise (RE) + functional exercise (FE)] in combination with, or without, a leucine-enriched whey protein isolate supplement on muscle strength, physical functioning, aerobic capacity, and cardiometabolic health in older adults (≥60 years). Physical activity levels were also evaluated 6 months post-cessation of the intervention.

**Methods:**

Forty-six, community-dwelling, previously untrained males, and females [age: 68 ± 5 years (mean ± SD); BMI: 27.8 ± 6.2 kg/m^2^] who completed the trial were initially randomized to one of two independent arms [Exercise *n* = 24 (E); Exercise+Protein *n* = 22 (EP)]. Both arms completed 16 weeks of RE (performed to fatigue) (2 times/week) with FE (1 time/week) on non-consecutive days. Additionally, EP were administered a leucine-enriched whey protein supplement (3 times/day) for 16 weeks based on individual body-weight (1.5 g/kg/day).

**Results:**

As a result of dietary supplementation, protein intake increased in EP (∼1.2 ± 0.4 to 1.5 ± 0.7 g/kg/day) during the intervention. Maximal strength (1RM) values for leg press (E: +39 ± 7 kg, *p* = 0.006; EP: +63 ± 7 kg, *p* < 0.001), chest press (E: +22 ± 4 kg, *p <* 0.001; EP: +21 ± 6 kg, *p* < 0.001), and bicep curl (E: +7 ± 0 kg, *p* = 0.002; EP: +6 ± 1 kg, *p* = 0.008) significantly increased in E and EP respectively, with no differences between arms (*p* > 0.05). Physical functioning in the obstacle course (E: -5.1 ± 6.8 s, *p* < 0.001; EP: -2.8 ± 0.8 s, *p* < 0.001) and short-physical performance battery scores (E: +0.5 ± 0.5, *p* = <0.001; EP: +0.4 ± 0.5, *p* = 0.038), and aerobic capacity in the 6-min walk test (E: +37 ± 24 m, *p =* 0.014; EP: +36 ± 3 m, *p* = 0.005) improved in E and EP respectively, with no differences between arms (*p* > 0.05). No significant change was observed for markers of cardiometabolic health (glycaemic control or blood pressure) (*p* > 0.05). At follow-up, 86% of older adults reported to performing physical activity ≥1 per week. Of those, 61% were still participating in strength- and cardiovascular- based exercise.

**Conclusion:**

Concurrent exercise (RE + FE) offers a potent method to combat age-related muscle weakness, and our results suggest a high proportion of older adults may continue to exercise unsupervised. However, leucine-enriched whey protein isolate supplementation did not confer any additional benefit in those already consuming ample amounts of dietary protein at trial enrolment. Future trials should utilize a whole-foods approach and investigate the effects in frail and non-frail older adults habitually consuming the RDA of protein, to assess if a higher intake of protein is needed to delay the onset of muscle weakness.

**Trial Registration:**

Clinicaltrials.gov Identifier: NCT02912130.

## Introduction

The aging epidemic has led to increased awareness of frailty phenotypes, notably muscle weakness (Fried et al., 2001), which manifests around 50 years of age, and occurs at a 2–5 times more rapid rate than muscle mass loss (Goodpaster et al., 2006). In the United Kingdom alone, estimated annual costs attributed to muscle weakness are $2.5 billion (Pinedo-Villanueva et al., 2018) which emphasizes the urgent need for prevention strategies.

Two prophylactics suggested to curtail muscle weakness are resistance exercise (RE) and dietary-protein. RE is a potent stimulus to increase muscle strength and physical functioning (Fiatarone et al., 1990; Stec et al., 2017) whilst epidemiological data show higher quantities of dietary-protein (>1 g/kg/day) can curb declines in grip strength (McLean et al., 2016) and mobility (Mustafa et al., 2018). Nonetheless, the body of evidence to support the increased requirement of dietary-protein to augment RE effects on muscle strength is inconclusive. Individual trials have failed to show benefits (Verdijk et al., 2009; Leenders et al., 2013; Holwerda et al., 2018) and only when trials are pooled in a meta-analysis does a positive effect appear (Cermak et al., 2012; Morton et al., 2017) although this has not always been the case (Finger et al., 2015; Gade et al., 2018). Disparate findings may be due to total amount, type and timing of supplemented protein, and in particular, sub-optimal intakes of the essential amino acid leucine, the key regulator of muscle anabolism (Devries et al., 2018). Acute trials utilizing isotope tracers have demonstrated an anabolic resistance in older adults, whereby higher dosages of dietary-protein rich in leucine are suggested to overcome this phenomenon (Moore et al., 2015).

Regarding the optimal intensity of RE, similar increases in strength have been evident when comparing moderate and heavy loads in the range of 40–90% of maximum (Morton et al., 2016; da Silva et al., 2018) once total volume is equated for, and lower loads are carried out to fatigue. Nevertheless, as 45.1% of 14,807 older adults (>75 years) suffer chronic musculoskeletal pain (Cimas et al., 2018) refraining from heavy repetitive loading may be a more practical choice to maintain adherence long term. Similar to RE intensity, comparable improvements in strength are apparent with two compared to three weekly sessions in older adults (Silva et al., 2017; Stec et al., 2017).

A Cochrane review (Sherrington et al., 2019) recently highlighted that combining multiple exercises (muscle strengthening, functional, and balance) offset falls in community-dwelling older adults by 34%. Considering this, in addition to the principle of specificity effect (Hawley, 2008; Reilly et al., 2009), there is strong evidence to include RE and functional exercise (FE) in a regimen to obtain the synergistic benefits on muscle strength and physical functioning. In addition, including FE may act as an added stimulus to confer cardiometabolic health benefits on blood pressure, glycaemic control, and aerobic capacity (Whitehurst et al., 2005; Pollock et al., 2018).

With the aforementioned research in mind, the aim of the present two-arm trial [Exercise (E); Exercise+Protein (EP)] was to investigate the synergistic effects of 16-weeks of RE (to fatigue) with FE, in combination with, or without, a leucine-enriched whey protein isolate supplement on muscle strength, physical functioning, and cardiometabolic health in older adults. It was hypothesized EP would demonstrate superior increases in muscle strength (our primary outcome) compared to E. Secondary aims included the effect of treatments on (a) physical functioning, (b) aerobic capacity, and (c) markers of cardiometabolic health which we anticipated to be superior in EP compared to E. Of tertiary interest was to examine physical activity levels 6 months post-cessation of the trial, which we envisaged to be low.

## Materials and Methods

### Subjects

Sample size was based on an average pooled effect size of 0.5 (range 0.1–0.9) from a previous meta-analysis (Cermak et al., 2012), which found greater increases in leg strength with combined RE and dietary-protein vs. RE alone in older adults. Using G^∗^Power (Faul et al., 2007) software and setting power to 80% with alpha at 0.05 (two-tailed) to observe a treatment effect *n* ≥ 32 participants were required for final analysis. Recruitment was conducted via online advertisement detailing trial information and enrolment was based on initial telephone screening outlining inclusion and exclusion criteria^1^. To confirm eligibility, participants completed a physical activity readiness questionnaire (PAR-Q) (Thompson et al., 2013) to screen for pre-existing medical conditions. During this time participants were briefed on the nature of the trial, associated risks and benefits before written informed consent was obtained. Participants were excluded with uncontrolled hypertension (160/100 mmHg), hypotension (≤100 mmHg), hyperglycaemia (HbA1c ≥ 10%), on prescribed hormonal and/or anti-inflammatory medication, previous history of scheduled exercise (past 12 months), recent musculoskeletal injury, intolerance to dairy and/or lactose products (for exhaustive list see text footnote 1). For the duration of the trial, participants were instructed to refrain from exercise, and/or nutritional supplements other than administered by the intervention. Ethical approval was sought from the North-West of England NHS Research Ethics Committee United Kingdom (REC No. 16/NW/0480) and the trial was registered at clinicaltrials.gov as NCT02912130.

### Trial Design

**FIGURE 1:**
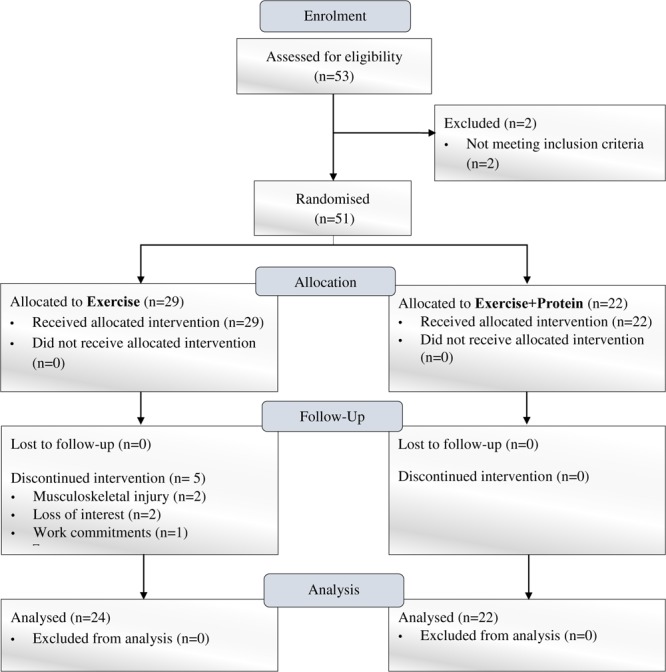
Flow chart of experimental trial.

Following enrolment, forty-six, non-frail, community-dwelling, and previously untrained males and females (aged ≥60–86 years) who completed the trial were initially randomized in a single-blind design to one of two independent arms [Exercise *n* = 24 (E); Exercise+Protein *n* = 22 (EP); see Figure 1]. All participants attended the clinical laboratories at two separate time points (pre- and post- intervention) where outcome measures were performed. During the intervention, E and EP attended the university sports complex gymnasium thrice weekly for one FE and two RE sessions (supervised by certified exercise trainers) on non-consecutive days for the duration of 16-weeks. EP were administered a leucine-enriched whey protein supplement thrice daily (at breakfast, lunch, and dinner) for 16 weeks based on individual body-weight. Protein supplements were consumed in addition to normal dietary intake. To minimize diurnal variation, the outcome measures were carried out at the same time of day pre- and post- intervention.

### Pre- and Post-outcome Measures

#### Anthropometry

Participants removed shoes, socks, watches, jewelry, and any heavy clothing prior to height (nearest 0.1 m; SECA 213 Stadiometer) and weight (nearest 0.1 kg; TANITA MC-180MA) measurements. Body mass index (BMI) was calculated from the above measurements using the following validated equation: body-weight (kg)/height (m^2^).

#### Muscle Strength

Strength was evaluated via 5-repetition maximum (RM) using established guidelines (Baechle and Earle, 2008) on the following exercises in orderly fashion: leg press, chest press, and bicep curl. Testing was performed on resistance machines (leg press and chest press) and with a barbell (bicep curl) using Technogym equipment at the university sports complex gymnasium. A ∼5 min low-intensity cardiovascular warm up was first conducted on either a motorized treadmill, cross-trainer, or bike. Lifting began with a self-selected moderate weight for 15 repetitions followed by 2 min rest before participants completed a further 10 repetitions with an increased weight selected by the exercise trainer. If full range of motion with correct posture was achieved the load was increased by 5 kg and 10 kg for upper- and lower- body, respectively. This process was continued with 2 min breaks until the true 5RM was obtained. 5RM values were then transformed to 1RM values using the previously validated equation (Brzycki, 1993) for strength testing in older adults (Wood et al., 2002). Final 1RM values in kilograms (kg) were used for analysis.

#### Physical Functioning and Aerobic Capacity

**FIGURE 2:**
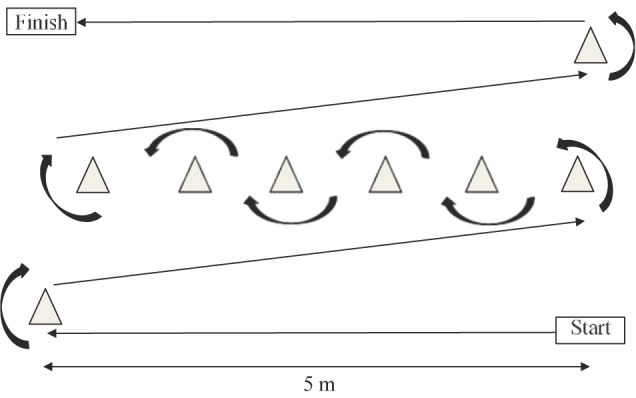
Mini obstacle course (25 m).

Standardized operating procedures were followed for the short-physical performance battery (SPPB) (Guralnik et al., 1994) which consisted of three timed components: standing balance, 4-m gait speed, and time to complete five chair-stands. Participant scores for each component were totalled between 0 and 12 used for analysis. The obstacle course was re-adapted from Steele et al. (2017) and consisted of a 25 m marked course incorporating 90 and 180 degree turns (Figure 2). Using a stopwatch to record time, participants were instructed to rise from the floor and carry a kettlebell weight (10 kg for males, 5 kg for females) as fast as possible around the course. The stopwatch stopped once the participant was re-seated on the floor at the finish line. Time in seconds (s) was used for analysis. For the 6 min walk test (6MWT) standardized operating procedures were followed (ATS Committee on Proficiency Standards for Clinical Pulmonary Function Laboratories et al., 2002). A 30 m track was marked in an environmentally controlled laboratory (17°C) with chairs placed at both ends. Participants were instructed to walk up and down the track covering as much ground as possible within 6 min. Participants were reminded the test was self-paced and if needed a rest was permitted; however, the stopwatch would continue to run. Participants completed two 6MWT with a 10 min break between tests, Average of the two distances in meters (m) was used for analysis.

#### Blood Pressure and Fasting Blood Samples

Participants were laying rested on a medical bed for ∼5 min prior to blood pressure measurement. An inflatable cuff (SphygmoCor^®^ CPV system; ScanMed Medical) was applied to the upper arm directly over the brachial artery and subsequent systolic and diastolic blood pressure readings (mmHg) were taken and used for analysis. A 35 μm capillary fingerstick blood sample was then collected in sterile conditions for the subsequent determination of plasma glucose (mmol/L; Alere Cholestech LDX Analyzer, Chesire, United Kingdom) and glycated hemoglobin (HbA1c %; Alere, Afinion^TM^, AS100, Cheshire, United Kingdom).

#### Physical Activity Follow-Up Survey

Six months post-cessation of the intervention all participants were re-contacted and asked to fill out an online survey (designed via Bristol Online Survey^2^). Survey was re-adapted from Forkan et al. (2006) and consisted of three multiple-choice questions. (1) How many times have you exercised in the past 4 weeks? (2) If you exercised in the past 4 weeks what type of exercise was it? (3) How long did each session last? Individual responses were totalled and analyzed to illustrate a %.

### Exercise Intervention

Participants completed a gym induction and attended a familiarization day where the correct range of motion for each RE exercise was demonstrated to ensure technique and minimize injury risk. Participants also practiced lifting the weight to fatigue (defined as the point where the weight could no longer be lifted with correct posture). Participants were provided with a booklet detailing weekly sessions, specific exercises, and shown how to track weights. For FE, participants were shown the correct movement for each exercise and familiarized with the Borg scale during a practice session. Session attendance was recorded on arrival at the gymnasium reception desk. Average attendance was totalled to give a %.

#### Resistance Exercise

Each sessions lasted ∼50 min; with 5 min warm up of low-intensity exercise on either a motorized treadmill, cross-trainer or bike, then continued with 45 min of whole-body REs. Participants first completed one upper- and lower- body warm up with a lightweight. Participants then self-selected a moderate weight and completed 2 sets to fatigue separated by 3 min/between sets and 3 min/between exercises on each of the following machines in orderly fashion: leg press, chest press, calf press, shoulder press, seated row, and back extension. Bicep curl was performed last using a free weighted barbell due to no machine-based option. Weight was increased for upper- and lower- body exercises by 2.5 and 5 kg, respectively, once the participant completed ≥12 repetitions in both working sets. Maximal effort and progressive overload was encouraged by the exercise trainer.

#### Functional Exercise

**FIGURE 3:**
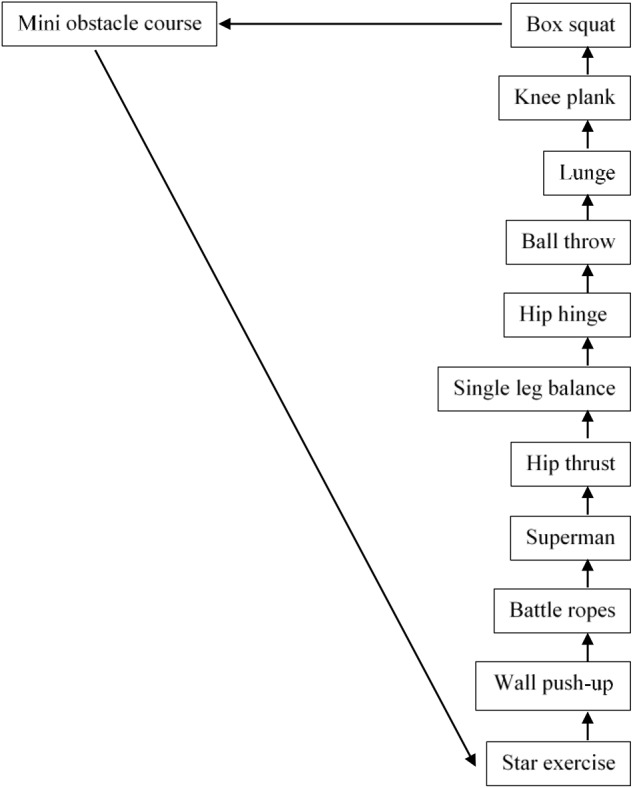
Functional exercise circuit.

Warm-up began with ∼10 min of low-intensity dancing to participants preferred choice of music. FE session consisted of 12 stations re-adapted from Whitehurst et al. (2005) with 1 min of exercise performed at each individual station before moving in order to the next. Each station was marked with the exercise station name, assigned a station number (between 1 and 12) and marked with a visible Borg CR-10 scale effort sheet. Participants completed the FE circuit 3 times with 3 min breaks between sets (see Figure 3). Participants were instructed to provide high effort throughout the session demonstrating a level of 7–10 on the Borg scale (Borg and Kaijser, 2006).

### Protein Supplementation and Dietary Control

All participants recorded their energy intake via 4-day food diaries pre- and post- intervention. Instructions were given how to correctly weigh food, measure liquids, and fill in the diaries. Protein supplements were weighed on scales (Weighstation Electronic Platform Scale, Devon, United Kingdom) and sealed in sachet bags (Tesco Stores, United Kingdom) according to participants’ individual body-mass (g/kg/body-weight). Participants in EP were administered a Vanilla flavored Whey Isolate Protein supplement (MyProtein, Northwich, Cheshire, United Kingdom) (at: 1.5 g/kg/day; 0.5 g/kg/meal) enriched with Leucine (MyProtein, Northwich, United Kingdom) (at: 0.09 g/kg/day; 0.03 g/kg/meal) and mixed with 200 ml of water which was ingested thrice daily (breakfast, lunch, dinner) for 16-weeks. This dosage has previously shown to overcome the anabolic resistance among older adults (Moore et al., 2015). Participants were reminded the protein supplement was to be consumed in addition to normal dietary intake. Adherence was assessed via self-report supplement logs and by counting returned sachets. Compliance with the protein supplement was totalled across the intervention to show a %.

### Statistical Analysis

Statistical analysis was performed using SPSS Statistics 24 (IBM Corporation, New York, United States). Food diaries were analyzed for energy and protein content through dietary analysis software (Nutritics LTD., Ireland). All data were checked for normality via Shapiro-Wilk test, which were violated for muscle strength and physical function measures. Percentage change and log transformations were unsuccessful at normalizing the data therefore non-parametric methods were utilized. Within-arm time effects (pre- and post- intervention) were analyzed by Wilcoxon-ranked paired tests. Between-arm differences (E vs. EP) were analyzed by Kruskal-Wallis (*H*) tests. Normality tests showed normal distribution for anthropometry, blood pressure, blood glucose, glycated hemoglobin, and food diary measures therefore parametric testing was utilized. Baseline comparisons were analyzed by students unpaired (*t*) tests. Independent arms were analyzed using a mixed model ANOVA with two arm levels (E vs. EP) and two time levels (pre- and post- intervention). If between arm effects were present they were followed up using Bonferroni *post hoc* comparisons. Mauchly’s test of sphericity was used to check homogeneity of variance; where necessary, any violations of the assumption were corrected using the Greenhouse–Geisser adjustment. Data are expressed as mean (±) standard deviation throughout. For descriptive purposes, percentage (±) is calculated from mean values. The alpha level for statistical significance was set at *p* < 0.05 *a priori*.

## Results

### Subjects

Participants included in the final analysis were distributed similarly in each arm and when split by gender no difference was detected (*p* = 0.55). Additionally, arms did not differ in any baseline measure (*p* > 0.05) (see Table 1).

**Table 1:** Baseline characteristics of participants.

Parameter	E	EP	*p* value
*n* = [number]	24	22	
Gender [male/female]	12/12	9/13	0.55
Age [years]	66 ± 4	69 ± 6	0.16
Height [m]	1.68 ± 0.1	1.64 ± 0.1	0.13
Weight [kg]	79.5 ± 21.6	74.2 ± 18.1	0.32
BMI [kg/m^2^]	28.1 ± 7.4	27.4 ± 4.9	0.63
Plasma glucose [mmol/L]	5.5 ± 0.6	5.4 ± 0.8	0.90
HbA1c [%]	5.5 ± 0.3	5.4 ± 0.3	0.67
Systolic blood pressure [mmHg]	142 ± 19	147 ± 17	0.36
Diastolic blood pressure [mmHg]	83 ± 16	82 ± 9	0.81
Leg press 1RM [kg]	131 ± 15	100 ± 48	0.06
Chest press 1RM [kg]	36 ± 16	36 ± 15	0.70
Bicep curl 1RM [kg]	19 ± 7	20 ± 6	0.58
SPPB [0–12]	11.5 ± 0.7	11.6 ± 0.7	0.31
Obstacle course time [s]	24.6 ± 12.3	22.0 ± 3.6	0.58
6MWT [m]	579 ± 83	582 ± 67	0.84
Energy intake [kcal/d]	1810.5 ± 385.7	1728.1 ± 359.5	0.55
Protein intake [kcal/d]	81.50 ± 27.1	77.26 ± 21.9	0.65
Protein intake [g/kg/day]	1.10 ± 0.4	1.16 ± 0.4	0.68
Protein intake [% total energy]	18 ± 4	18 ± 3	0.96
Total carbohydrate intake [g/day]	191.81 ± 40.2	168.8 ± 41.5	0.14
Total carbohydrate intake [% total energy]	43 ± 6	39 ± 6	0.11
Total fat intake [g/day]	69.75 ± 18.4	69.56 ± 23.1	0.98
Total fat intake [% total energy]	35 ± 6	36 ± 7	0.70

*Data are shown as means ± standard deviations. No significant differences were detected between baseline treatments (E, exercise; EP, exercise + protein) (*p* > 0.05). HbA1c, glycated hemoglobin; 1RM, 1 repetition maximum; SPPB, short physical performance battery; 6MWT, 6-min walk test.*

### Exercise and Dietary Adherence

Participants in E and EP attended 77 ± 10% and 78 ± 10% of their prescribed exercise sessions, respectively. A lower degree of compliance was observed with dietary- protein supplementation: EP = 43 ± 14%. As a result of supplementation, protein intake increased from ∼1.2 ± 0.4 at baseline to 1.5 ± 0.7g/kg/day in EP during the intervention period.

### Effect of Intervention

#### Anthropometry, Blood Pressure, and Blood Measures

No within- or between- arm differences were observed for height, weight, BMI, blood pressure, plasma glucose or glycated hemoglobin (*p* > 0.05) (Table 2). Although minor (non-significant) decreases in systolic blood pressure (E: 142 ± 19 to 137 ± 13, -5 mmHg; EP: 147 ± 17 to 143 ± 17, -4 mmHg) were evident from pre- to post-intervention in E and EP, respectively.

**Table 2:** Effect of intervention on anthropometry, blood pressure, and blood measures.

Parameter	E			EP		
	Pre	Post	Time *p*	Pre	Post	Time *p*	Time^∗^group
Height [m]	1.68 ± 0.1	1.68 ± 0.1	1.000	1.64 ± 0.1	1.64 ± 0.1	1.000	0.302
Weight [kg]	79.5 ± 21.6	78.7 ± 19.8	0.309	74.2 ± 18.1	73.6 ± 17.5	0.970	0.374
BMI [kg/m^2^]	28.1 ± 7.4	27.8 ± 6.6	0.319	27.4 ± 4.9	27.3 ± 4.5	0.977	0.379
Plasma glucose [mmol/L]	5.5 ± 0.6	5.5 ± 0.8	0.852	5.4 ± 0.8	5.4 ± 0.8	0.516	0.576
HbA1c [%]	5.5 ± 0.3	5.5 ± 0.4	0.339	5.4 ± 0.3	5.5 ± 0.3	0.378	0.821
Systolic pressure [mmHg]	142 ± 19	137 ± 13	0.258	147 ± 17	143 ± 17	0.329	0.894
Diastolic pressure [mmHg]	83 ± 16	82 ± 8	0.413	82 ± 9	83 ± 9	0.810	0.414

*Values are means ± standard deviations. No significant differences between treatments (E, exercise; EP, exercise + protein) (*p* > 0.05). HbA1c, glycated hemoglobin.*

#### Muscle Strength

**FIGURE 4:**
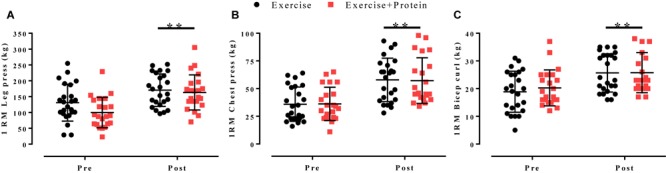
1RM values for **(A)** leg press, **(B)** chest press, and **(C)** bicep curl in response to independent treatments (E, black; EP, red). Individual data points are shown with horizontal line indicating the mean and error bars representing the standard deviation. Within-arm time effects were evident post-intervention for leg press, chest press, and bicep curl all (^∗^*p* < 0.05). No between-arm effects observed (*p* > 0.05).

Following 16 weeks of progressive resistance and FE 1RM values for leg press (E: 131 ± 58 to 170 ± 51 kg, +30%, *p* = 0.006; EP: 100 ± 48 to 163 ± 55 kg,+63%, *p* < 0.001), chest press (E: 36 ± 16 to 58 ± 20 kg, +60%, *p <* 0.001; EP: 36 ± 15 to 57 ± 21 kg, +58%, *p* < 0.001) and bicep curl (E: 19 ± 7 to 26 ± 7 kg,+37%, *p* = 0.002; EP: 20 ± 6 to 26 ± 7 kg, +30%, *p* = 0.008) significantly increased from pre- to post-intervention in E and EP, respectively. However, no between-arm differences were observed(*p* > 0.05; Figure 4).

#### Physical Functioning and Aerobic Capacity

Time to complete the obstacle course (E: 24.6 ± 12.3 to 19.5 ± 5.5 s, +21%, *p* < 0.001; EP: 22.0 ± 3.6 to 19.2 ± 4.1 s, +13%, *p* = *p* < 0.001), performance in the SPPB (E: 11.5 ± 0.7 to 12.0 ± 0.2points, +4%, *p* = <0.001; EP: 11.6 ± 0.7 to 12.0 ± 0.2 points, +3%, *p* = 0.038) and aerobic capacity in 6MWT (E: 579 ± 83 to 616 ± 107 m, +6%, *p =* 0.014; EP: 582 ± 67 to 618 ± 64 m, +6%, *p* = 0.005) significantly improved from pre- to post-intervention in E and EP, respectively. No between-arm differences were observed (*p* > 0.05; Table 3).

**Table 3:** Effect of intervention on physical function and aerobic capacity.

Parameter	E			EP		
	Pre	Post	Time *p*	Pre	Post	Time *p*	Time^∗^group
SPPB [1–12]	11.5 ± 0.7	12.0 ± 0.2	<0.001	11.6 ± 0.7	12.0 ± 0.2	0.038	0.924
Obstacle course time [s]	24.6 ± 12.3	19.5 ± 5.5	<0.001	22.0 ± 3.6	19.2 ± 4.1	<0.001	0.930
6MWT [m]	579 ± 83	616 ± 107	0.014	582 ± 67	618 ± 64	0.005	0.974

*Values are means ± standard deviations. No significant differences between treatments (E, exercise; EP, exercise + protein) (*p* > 0.05). SPPB, short physical performance battery; 6MWT, 6-min walk test.*

#### Physical Activity Levels: Post-trial Follow-Up

**FIGURE 5:**
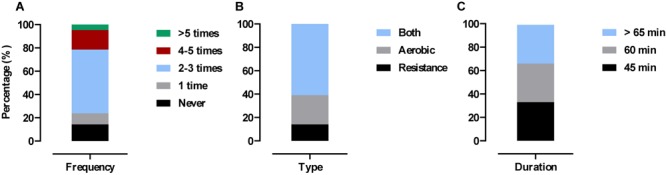
Physical activity levels 6 months post-trial. Values are presented as pooled responses (%) from survey questions. **(A)** How many times have you exercised in the past 4-weeks? **(B)** If you exercised in the past 4-weeks what type of exercise was it? **(C)** If you exercised in the past 4-weeks how long did each session last?

Forty-two out of 46 participants completed the 6-months post-trial physical activity survey. No significant differences were observed between arms for any survey question (*p* > 0.05). Pooled results showed 86% (36/42) were still exercising at least 1/week with 14% (6/42) not exercising. Of those subjects still exercising 25% (9/36) reported to performing aerobic exercise (cardiovascular based, i.e., walking, cycling, jogging, swimming, and yoga), 14% (5/36) reported performing RE (weight-bearing, i.e., lifting weights, body-weight exercises) and 61% (22/36) reported to performing both. The duration of these exercise sessions varied between 45 min (33%) (12/36), 60 min (33%) (12/36), and >60 min (33%) (12/36) (see Figure 5).

## Discussion

We report 16 weeks of progressive resistance and FE (3 times/week) significantly improved muscle strength, physical functioning, and aerobic capacity without influencing blood pressure or glycaemic control in previously untrained older adults. In addition, leucine enriched-whey protein supplementation (3 times/day) did not confer any additional benefit on these outcomes.

We primarily sought to investigate if leucine enriched-whey protein supplementation would augment muscle strength during combined exercise training in older adults. Following recommendations (Paddon-Jones and Rasmussen, 2009; Bauer et al., 2013) we provided ample amounts of dietary-protein (0.5 g/kg/meal) enriched with leucine (0.03 g/kg/meal; >3 g per serving) thrice daily to maximize the muscle protein synthetic response (Moore et al., 2015). Despite substantial increases in muscle strength (Figure 4) and physical/aerobic performance (Table 3) we observed no difference between treatments. This finding is in line with existing data (Kukuljan et al., 2009; Verdijk et al., 2009; Leenders et al., 2013; Stragier et al., 2016; Holwerda et al., 2018) which failed to show a synergistic effect of RE and dietary-protein in strength among community-dwelling older adults. Similar to the above trials, our population of older adults were non-frail i.e., demonstrated high baseline SPPB (11.5 ± 0.7) and 6MWT (583 ± 75) scores. In contrast, benefits have been observed in pre-frail/functionally impaired older adults with lower habitual levels of dietary-protein (Cawood et al., 2012; Tieland et al., 2012). Thus, the relative good health of our population who were habitually consuming adequate amounts of dietary-protein (∼1.2 ± 0.4 g/kg/day) may have masked any effect of supplementation (Table 4). Despite increasing dietary-protein intake from ∼1.2 ± 0.4 to 1.5 ± 0.7 g/kg/day during the present trial, adherence (43 ± 14%) was considerably lower than others (Verdijk et al., 2009; Bell et al., 2017) although similar in those attempting to supplement 3 times/day (Norton et al., 2016). Considering this, coupled with the undesirable verbal feedback relating to supplement taste we recommend future trials use a whole-food approach to increase palatability and adherence as previously described (Haub et al., 2002; Wright et al., 2018).

**Table 4:** Dietary-intake from self-recorded 4-day food diaries.

Parameter	E			EP		
	Pre	Post	Time *p*	Pre	Post	Time *p*	Time^∗^group
Energy intake [kcal/d]	1810.5 ± 385.7	1944.1 ± 568	0.282	1728.1 ± 359.5	1969.3 ± 429.9	0.012	0.454
Protein intake [kcal/d]	81.50 ± 27.1	77.63 ± 20.5	0.512	77.26 ± 21.9	109.61 ± 30.8^∗^	<0.001	0.002
Protein intake [g/kg/body mass/day]	1.10 ± 0.4	1.04 ± 0.3	0.361	1.16 ± 0.4	1.63 ± 0.5^∗^	<0.001	<0.001
Protein intake [% total energy]	18 ± 4	16 ± 3	0.193	18 ± 3	23 ± 6^∗^	0.004	0.003
Total carbohydrate intake [g/day]	191.81 ± 40.2	211.09 ± 68.3	0.674	168.8 ± 41.5	187.8 ± 59.7	0.202	0.989
Total carbohydrate intake [% total energy]	43 ± 6	44 ± 6	0.760	39 ± 6	38 ± 7	0.292	0.398
Total fat intake [g/day]	69.75 ± 18.4	72.96 ± 21.7	0.174	69.56 ± 23.1	75.49 ± 24.4	0.165	0.744
Total fat intake [% total energy]	35 ± 6	34 ± 5	0.719	36 ± 7	34 ± 7	0.396	0.688

*Values are means ± standard deviations. ^∗^Indicates between-arm difference post-intervention (*p* < 0.05).*

All strength measures improved from pre- to post- intervention by >30% (Figure 4) adding to the current body of research (Charette et al., 1991; Latham et al., 2004; Nilwik et al., 2013; Bell et al., 2017) demonstrating prolonged resistive exercise modalities (≥12 weeks) are a potent method to combat age-related muscle weakness. Together, these data offer an alternative approach for older adults who may be reluctant to use heavy loads due to health or personal constraints.

The observed increases in strength were accompanied by a favorable shift in physical functioning and aerobic capacity (Table 3). Whilst difficult to distinguish which part of the multifaceted exercise regimen contributed specifically to these improvements, each may have played a complementary role. For instance, RE increases in strength can improve SPPB performance (Tieland et al., 2012, 2015) whereas FE may have predominately enhanced mobility on the obstacle course (Rosendahl et al., 2008) and provided that added stimulus to increase endurance on the 6MWT (Whitehurst et al., 2005). In support, three studies (Arnarson et al., 2013; Kawada et al., 2013; Oesen et al., 2015) found no effect of RE on 6MWT distance, whilst in the present trial and in others (Bell et al., 2017) combining RE with endurance elements of training resulted in improved 6MWT distance. It is difficult to elaborate further as it was not the purpose of the trial to compare these exercise modalities, and associations between neuromuscular attributes and performance indices are not fully understood (Jacob et al., 2018). Nonetheless, the above findings are clinically relevant considering muscle strength declines at an annual rate of ∼2–3% after the fifth decade of life (Goodpaster et al., 2006) and is adversely characterized by reductions in functional capacity (Pavasini et al., 2016), and activities of daily living (Rantanen et al., 2002).

Our multifaceted exercise regimen was designed to optimize muscle strength, physical functioning, aerobic capacity and metabolic health all of which deteriorate with age (Pendergast et al., 1993; Niccoli and Partridge, 2012). Regarding the latter, we failed to observe a change in markers of cardiometabolic health (Table 2) which is in contrast to others (Bell et al., 2017) employing combined strength and high-intensity interval exercise. Thus, we postulate the lack of adaptation in glycaemic control/blood pressure may be due to an insufficient intensity of the exercise regimen employed, or alternatively, due to a lack of reduction in body-weight which may have concealed alterations.

Exercise adherence was high (78 ± 10%) across the 16-week intervention period and was even higher during follow-up (6 months post-intervention) with 86% (36/46) of previously untrained older adults reporting to performing physical activity ≥1 per week (Figure 5). Of those, 61% (22/36) were participating in strength- and cardiovascular- based exercise which aligns with current exercise recommendations for older adults (Nelson et al., 2007). The above figures are promising considering older adults are highlighted as the least active section of society with astonishingly low numbers (<5%) meeting guidelines (Davis et al., 2011; Loustalot et al., 2013; Sun et al., 2013; Van Holle et al., 2014; Dalbo et al., 2015). By continuing to perform concurrent exercise our older adults are inevitably reducing the risk of age-related disease (Vellas et al., 2018) and mortality (García-Hermoso et al., 2018). Even slight increases in RE participation rates (as achieved here) may significantly relieve the economic burden of aging as costs attributed to muscle weakness are estimated at an annual $2,707 per person in the United Kingdom alone (Pinedo-Villanueva et al., 2018).

### Limitations

A clear drawback of our trial was the lack of compliance (43 ± 14%) to dietary-protein supplementation. As mentioned, future research should use a whole-food approach as greater adherence rates (>90%) have been evident (Haub et al., 2002; Wright et al., 2018). Another perceived limitation may relate to our population of older adults who were non-frail. By incorporating frail older adults, perhaps greater effects of treatments may have been observed. However, as mounting commentary (Paddon-Jones and Rasmussen, 2009; Bauer et al., 2013) advocate higher dietary-protein intakes (≥1.2 g/kg/day) for older adults it would be unwise to examine the effects in functionally impaired populations alone. For public health mandates to endorse a greater intake of dietary-protein above the current RDA (0.8 g/kg/day); evidence needs to be established across various populations (i.e., in community-dwelling and institutionalized older adults).

## Conclusion

To conclude, 16 weeks of progressive resistance and FE (3 times/week) significantly improved muscle strength, physical functioning and aerobic capacity without affecting blood pressure or glycaemic control in previously untrained older adults. In addition, leucine-enriched whey protein supplementation (3 times/day) did not yield further benefits. Nonetheless, 86% (42/46) of older adults were still performing strength- and cardiovascular- based exercise 6-months post-trial demonstrating clinical relevance. Finally, future research should focus on methods to incorporate high dietary-protein intakes (∼1.5 g/kg/day) through naturally occurring food sources in frail and non-frail older adults habitually consuming the RDA of protein. In turn, this may improve adherence rates and enable the efficacy of combined RE with dietary-protein on muscle strength to be evaluated.

## Author Contributions

BK, KM, FA, and OK have made substantial contributions to the trial design, data collection and interpretation, and are fully conversant with its content. BK wrote the full manuscript.

## Conflict of Interest Statement

The authors declare that the research was conducted in the absence of any commercial or financial relationships that could be construed as a potential conflict of interest.

## Footnotes

1 https://clinicaltrials.gov/ct2/show/NCT02912130

2 https://admin.onlinesurveys.ac.uk
